# Omicron SARS-CoV-2 variant of concern: A review on its transmissibility, immune evasion, reinfection, and severity

**DOI:** 10.1097/MD.0000000000029165

**Published:** 2022-05-20

**Authors:** Md Mohsin, Sultan Mahmud

**Affiliations:** a Applied Statistics, Institute of Statistical Research and Training, University of Dhaka, Dhaka, Bangladesh; b International Centre for Diarrhoeal Disease Research, Bangladesh (ICDDR’B), 68 Shaheed Tajuddin Ahmed Ave, Dhaka, Bangladesh.

**Keywords:** Covid-19, delta, immune evasion, omicron, reinfection, severity, transmissibility

## Abstract

Omicron, the new Covid-19 variant, has already become dominant in many countries and is spreading at an unprecedented speed. The objective of this study was to review the existing literature on Omicron's transmissibility, immune evasion, reinfection, and severity.

A literature search was performed using “PubMed,” “Web of Science,” “Scopus,” “ScienceDirect,” “Google Scholar,” “medRxiv,” and “bioRxiv.” Data were extracted from articles that reported at least one of the following: transmissibility, immune evasion, reinfection, and severity related to Omicron.

We found that Omicron spread faster than any other variant. This higher transmissibility can be ascribed to its extraordinary ability to evade the immunity developed by both vaccination and previous infections. However, we found that infections by Omicron were significantly less severe than those caused by Delta and other previous variants. We observed a significantly lower incidence of hospitalization, intensive care unit admission, and mechanical ventilator use in Omicron infections than in Delta or other variants. A substantially shorter median hospital stay and lower fatality rate were also observed in the Omicron infections. Despite Omicron's higher potential to evade immunity, vaccines and booster shots were found to be still significantly effective in protecting against severe Covid-19 infections.

Omicrons may be less severe than other variants of concern. However, its immune evasiveness and rapid spread pose an enormous threat to the global healthcare system.

## 1 Introduction

The WHO identified B.1.1.529 as a variant of concern on November 26, 2021, following advice from the World Health Organization Technical Advisory Group on Virus Evolution.^[1]^ The variant first detected in South Africa was named Omicron. The variant stood out because it involves more than 30 mutations to the spike protein of the SARS-CoV-2, which detects host cells and is the primary target of the body's immune responses.^[2]^ Omicron is a highly divergent variant due to its many mutations, which are likely associated with humoral immune evasion and increased transmissibility.^[3]^ For various reasons, the total risk associated with Omicron remains exceptionally high. First, COVID-19 poses a significant worldwide threat. Second, recent evidence suggests that Omicron surpasses other variants (Delta) in terms of proliferation, resulting in a rapid community spread.^[3]^ The rapid increase in cases may result in an increase in hospitalizations, place a strain on health care systems, and result in severe morbidity, particularly among vulnerable populations.

Rapid transmission of the Omicron variant generated the fourth wave of SARS-CoV-2 infections in South Africa, with daily diagnosed illnesses exceeding the total recorded in the country during all preceding periods.^[4]^ As of January 2022, the Omicron variant has spread widely around the world, including the United States, where the Omicron variant was believed to be responsible for 95% of all SARS-CoV-2 illnesses detected during the week ending January 1, 2022.^[5]^

The overall threat posed by Omicron is highly dependent on four critical factors:

1. how transmissible the variant is;
2. how well vaccines and prior infections protect against infection, transmission, clinical disease, and death;
3. the virulence of the variant in comparison to other variants; and
4. how well populations recognize these dynamics, perceive risk, and adhere to control measures, including public health and social measures.^[3]^

Therefore, extensive research is required to answer these questions. To date, very few studies have been conducted on the transmissibility, immune evasion, reinfection, or severity of the Omicron variant. Therefore, this study aimed to review the existing literature on the transmissibility, immune evasion, reinfection, and severity of the Omicron variants compared to other variants.

## 2 Method

A literature search was performed in “PubMed,” “Web of Science,”“ Scopus,” “ScienceDirect,” “Google Scholar,” “medRxiv,” and “bioRxiv” using the following key words: “SARS-CoV-2,” “coronavirus,” “COVID-19,” “Omicron,” “Transmissibility,” “Immune Evasion,” “Reinfection,” and “Severity” to find articles published in 2021 and 2022. We checked the reference lists of all the studies identified using the above methods. Data were extracted from articles that reported at least one of Omicron's transmissibility, immune evasion, reinfection, or severity. Since this is a review study, no ethical approval is required.

## 3 Review findings

### 3.1 Transmissibility

On January 10, 2022, the U.S reported a record 1.35 million new coronavirus infections, almost 5 times higher than the peak points of all other waves.^[6]^ Similar patterns have been observed in many European countries.

In Norway, a study found that among 117 participants in a party, 74% were infected with Omicron. In the party, there was 1 participant from South Africa (the country where Omicron was first detected and later spread).^[7]^ Among the partygoers, about 96% were fully vaccinated. The study concluded that Omicron is highly contagious, even among vaccinated people.

Researchers in Denmark analyzed the transmission of Omicron and Delta variants among household members (11,937 households) and determined that Omicron was approximately 2.6 times (95% CI: 2.34–2.90) to 3.7 times (95% CI: 2.65–5.05) more infectious than the Delta variant among vaccinated and boosted individuals.^[8]^ However, among unvaccinated people, Omicron was only 1.17 times (95% CI: 0.99–1.38) more infectious than Delta, which was statistically insignificant.

### 3.2 Immune evasion and reinfection

The most significant issue regarding the new variant is whether it can evade the immunity developed by vaccinations and previous infections. Several studies have been found in the literature that inspected the evasion ability of Omicron compared to other variants. Between November 30, 2021, and January 1, 2022, one study in the United States investigated clinical and epidemiologic data from cases testing positive for SARS-CoV-2 infection inside the Kaiser Permanente Southern California healthcare system. The study included 52,297 cases of S-Gene Target Failure (SGTF) (Omicron) infection and 16,982 cases of non-SGTF (Delta [B.1.617.2]) infection.^[9]^ The adjusted odds of having previously been infected with SARS-CoV-2 were 4.45 (95% CI: 3.24–6.12) fold greater in individuals with Omicron variant infections than in those with Delta variant infections. Likewise, the adjusted odds of receiving any vaccine series (1, 2, or 3 doses of BNT162b2/mRNA-1973 or Ad.26.COV2.S with or without a booster dose of any vaccine) were significantly greater (2–6 times) in cases with Omicron variant infections than in cases with Delta variant infections.

In a large-scale study in Denmark, Lyngse et al found that among vaccinated people, Omicron was 2.6–3.7 times more infectious than the Delta variant. However, they found no significant difference in transmissibility between the 2 variants in unvaccinated people.^[8]^ They summarized that the rapid spread of Omicron Variant of Concerns can be attributed to their immune evasiveness, rather than an inherent increase in fundamental transmissibility.

Pullium et al conducted a study in South Africa with routine surveillance data of 2,796,982 persons who tested positive for SARS-CoV-2 in a laboratory at least 90 days prior to November 27, 2021, to examine the risk of reinfection. The analysis found that, in contrast to the Beta and Delta, the Omicron variant of SARS-CoV-2 demonstrates substantial population-level evidence for evasion of immunity from prior infection.^[10]^

Reduced neutralization of the Omicron variant has been observed in investigations using plasma specimens from individuals who received the entire (two-or three-dose) mRNA vaccination series^[11]^ and from patients who had previously been infected with SARS-CoV-2.^[12]^ In addition, several early observational studies revealed that COVID-19 vaccinations were substantially less efficacious in preventing Omicron variant infections.^[13–15]^

Researchers at the University of Edinburgh, UK, found that the rate of possible reinfection for the Omicron variant was approximately 10 times that of the Delta variant.^[16]^ Also, Brandal et al demonstrated that Omicron has a more remarkable ability to evade the immunity developed by vaccines. They showed that 74% of people got infected in a party where 96% of the participants were fully vaccinated against SARS-CoV-2.^[7]^ In a national database study in Qatar, Altarawneh et al found lower protection by the previous infection against the Omicron variant.^[17]^

### 3.3 Severity

The severity of Covid-19 is measured based on the requirements of hospital admission, length of hospital stay, requirement of ventilators, time required to recover, and mortality. There is some unanimity regarding the severity of the new Omicron variant found in existing literature. The Southern California study that included 52,297 cases of SGTF (Omicron) infection and 16,982 non-SGTF (Delta) infections reported a significantly decreased risk of severe clinical outcomes and shorter length of hospital stay.^[9]^ Hospitalizations for Omicron and Delta variant infections occurred in 235 (0.5%) and 222 (1.3%) cases, respectively. Throughout the follow-up period, no instances of Omicron variant infection required mechanical ventilation, compared to 11 cases of Delta-variant infection (*P* < .001). In addition, the median length of stay in the hospital was 3.4 (2.8–4.1) days shorter for hospitalized individuals with Omicron variant infections than for hospitalized cases with Delta variant infections, indicating a 70% (95% CI: 64.0–74.5%) reduction in hospital length of stay.

Researchers in South Africa assessed the clinical severity of the SARS-CoV-2 Omicron variant using nationwide data. After adjusting for confounding variables, they found that individuals with Omicron (SGTF) infection had an 80% lower risk of hospitalization than those without Omicron infection (aOR 0.2, 95% CI: 0.1–0.3).^[4]^ Moreover, compared with earlier Delta infections, after adjusting for risk factors for severe disease, those infected with Omicron had a 70% decreased risk of severe disease (aOR 0.3, 95% CI: 0.2–0.5).

The study in Scotland conducted by researchers from the University of Edinburgh indicated that, when compared to Delta, Omicron is related to a two-thirds reduction in the probability of COVID-19 hospitalization.^[16]^ They also found that while vaccination provides the best protection against Delta, the third/booster dose provides significant protection against the risk of symptomatic COVID-19 infection in Omicron when compared to ≥25 weeks after the second vaccine dose.^[16]^

The clinical characteristics of 466 patients infected with the Omicron variant admitted to a large hospital in Tshwane, South Africa, were compared to those of 3962 hospital admissions from earlier waves. Deaths and ICU admissions were 4.5% versus 21.3% (*P* < .0001) and 1% versus 4.3% (*P* < .0001) for the Omicron and preceding waves, respectively; length of stay was 4.0 days versus 8.8 days.^[18]^

A study conducted in England observed a reduction in the risk of hospitalization for Omicron infections compared to Delta infections during the study period (December 1 to December 14, 2021). The magnitude of the reduction varied according to the inclusion criteria for cases and hospitalization, ranging from 20% to 25% when any hospitalization was used as the endpoint to 40% to 45% when hospitalizations lasting one day or more were used.^[19]^

A retrospective cohort study (Omicron and Delta cohorts) was performed using electronic health record (EHR) data from 577,938 patients with first-time SARS-CoV-2 infection in the United States.^[20]^ After adjustment for demographics, socioeconomic determinants of health, comorbidities, medications, and immunization status, the 3-day risks in the Emergent Omicron cohort were consistently less than half of those in the Delta cohort: Emergency Department (ED) visit: 4.55% versus 15.22% (risk ratio or RR: 0.30, 95% CI: 0.28–0.33); hospitalization: 1.75% versus 3.95% (RR: 0.44, 95% CI: 0.38–0.52]); ICU admission: 0.26% versus 0.78% (RR: 0.33, 95% CI:0.23–0.48); and mechanical ventilation: 0.07% versus 0.43% (RR: 0.16, 95% CI: 0.08–0.32).^[20]^

Hay et al studied the viral dynamics and duration of PCR positivity of the SARS-CoV-2 Omicron variant in the United States using combined anterior nares and oropharyngeal samples of 10,324 individuals from the National Basketball Association's (NBA) occupational health program.^[21]^ Omicron infections lasted 9.87 days on average (95% CI: 8.83–10.9) compared to 10.9 days (95% CI 9.41–12.4) for Delta infections. In addition, the peak viral RNA based on cycle threshold (CT) values was lower for Omicron infections than for Delta infections (CT 23.3, 95% CI: 22.4–24.3 for Omicron; CT 20.5, 95% CI: 19.2–21.8 for Delta) and the clearance phase was shorter for Omicron infections (5.35 days, 95% CI: 4.78–6.00 for Omicron; 6.23 days, 95% CI: 5.43–7.17 for Delta).^[21]^

## 4 Discussion

Amidst an unprecedented surge in Covid-19 cases globally, presumably due to the emergence of the new variant Omicron, we conducted a rapid review of existing knowledge on the variant's transmissibility, immune evasion, reinfection, and severity. We found that the Omicron variant could spread faster than any other previous variants. However, researchers argue that faster transmission might occur because of Omicron's strong ability to evade immune responses induced by vaccinations and previous infections.^[8]^ In this rapid review study, we found that Omicron has several times higher odds of infecting fully vaccinated and previously infected people than Delta and other variants, which is consistent with other studies.^[7,9,11,12,16]^ Another study also found no significant difference in the risk of infections among unvaccinated people when they compared the Omicron variant with Delta.^[8]^

Although Omicron showed a greater ability to evade immunity developed by vaccinations and previous infections, we found a significantly reduced severity of Omicron infections. Almost all the studies we reviewed consistently reported a considerably lower risk of requiring hospital admission, ICU and mechanical ventilators, shorter median stay at the hospital, and lower mortality rates among Omicron infections compared to Delta and other variants.^[4,9,16,18–20]^ In addition, we found that although Omicron has a greater ability to escape immunity developed by vaccines, booster/third doses are still significantly effective in protecting against symptomatic Covid-19 infections.^[16,21,22]^

A team of researchers in Hong Kong compared the replication competency and cellular tropism of all Covid variants in ex vivo explant cultures of the human bronchus and lungs. They demonstrated that Omicron replicated more rapidly in the bronchus than any other SARS-CoV-2 strain but less efficiently in the lung parenchyma.^[23]^ This finding may be a probable reason for the reduced severity of Omicron infections. Identical findings were found in an animal trial as well.^[24]^

This timely review study accumulated the available information on Omicron, a new emerging variant of Covid-19. It summarizes quantitative data on popular questions on Omicron: transmissibility, immune evasion, reinfection, and severity. However, this study had some limitations. There is a scarcity of literature on the new variant of Covid-19. All the studies in our data are based only on Europe, America, and South Africa. Importantly, most of the studies cited in this review have not yet been peer reviewed.

Our review concludes that Covid-19 infections associated with the Omicron variant are less severe than those associated with other variants of concern. However, the findings of Omicron variant infections being more transmissible and immune evasion following prior infection and vaccination are concerning.^[9]^ The rapid spread of the Omicron variant over a short period of time has resulted in extraordinary Covid-19 outbreaks in some parts of the world. Even though Omicron is less severe than non-Omicron variants, due to high infection rates and immune escape, Omicron might overburden healthcare systems worldwide. The usual prevention methods, such as vaccination, masking, and suitable infection mitigation strategies are highly recommended for curtailing transmission, reducing morbidity and death, and easing the load on health systems worldwide. However, the findings of this study should be interpreted with caution because of the lack of peer-reviewed content the study is based on.

## Author contributions

**Conceptualization:** Md Mohsin, Sultan Mahmud.

**Methodology:** Md Mohsin, Sultan Mahmud.

**Writing – original draft:** Md Mohsin, Sultan Mahmud.

**Writing – review & editing:** Md Mohsin, Sultan Mahmud.
